# Trigeminal Numbness After Intracranial Repair of a Spontaneous Meningoencephalocele of the Lateral Wall of the Sphenoid Sinus

**DOI:** 10.7759/cureus.16026

**Published:** 2021-06-29

**Authors:** Orlando De Jesus, Allyson Pagán-Rodríguez, George Santiago Quiñones, Juan L Pérez-Berenguer

**Affiliations:** 1 Neurosurgery, University of Puerto Rico, Medical Sciences Campus, San Juan, PRI; 2 Pathology and Laboratory Medicine, University of Puerto Rico, Medical Sciences Campus, San Juan, PRI

**Keywords:** encephalocele, hypoesthesia, meningoencephalocele, numbness, sphenoid sinus, trigeminal nerve

## Abstract

A 58-year-old female with an eight-year history of rhinorrhea and a two-day history of subjective fever, chills, and vomiting presented to the emergency department for neurosurgical evaluation. Brain MRI demonstrated herniation of the meninges and portions of the inferomedial right temporal lobe through a defect of the lateral wall of the right sphenoid sinus, extending to the sphenoethmoidal recess and posterior right ethmoid air cells. A right pterional craniotomy was performed where the herniated part of the right temporal lobe, and its associated meninges, were excised. After surgery, she had hypoesthesia at the right maxillary division of the trigeminal nerve. This finding was caused by the proximity of the trigeminal nerve to the dural dissection that we performed at the bone defect. This rare complication has never been described after intracranial surgery. Only eight literature reports have described hypoesthesia or paresthesia of the trigeminal nerve after endoscopic resection of a sphenoid sinus meningoencephalocele. The patient has not had any recurrence of rhinorrhea after a six-month follow-up period.

## Introduction

Meningoencephaloceles are herniation of the meninges and parts of the brain through defects in the cranial vault. Obesity and female sex are risk factors for its development. Lateral sphenoid wall meningoencephaloceles frequently present with a cerebrospinal fluid (CSF) leak. However, misdiagnosis can occur, which can jeopardize the patient. Some patients can present with trigeminal nerve symptoms of numbness or paresthesias. Surgical planning is individually tailored to each patient, according to their specific defects. Postoperative complications directly associated with the resection of the meningoencephalocele are rare; however, rare cases of trigeminal numbness or paresthesias have been described after endoscopic repair. This complication has not been reported after intracranial repair.

## Case presentation

A 58-year-old female with no known drug allergies presented to the emergency department with an eight-year history of waxing and waning headaches and rhinorrhea and a two-day history of subjective fever, chills, and vomiting. Her past medical history was significant for morbid obesity (body mass index 45.5 kg/m^2^) and hypertension. She denied any significant head trauma. Physical exam showed a cooperative female without any gross focal neurologic deficits or any appreciable hemodynamic, respiratory, or psychiatric distress. She did not present any signs of meningeal irritation. Visual acuity was 20/30 bilateral, with intact visual fields by confrontation. The funduscopic examination did not have evidence of papilledema. No rhinorrhea was observed while the patient was in the supine position; however, it was appreciated when asked to sit up and bend forward at a rate of around one drop every 25 seconds. She noted that this frequency was much less than what she was accustomed to. Initial vital signs were significant for hypertension (174/96 mmHg), which was corrected with medications. Laboratory studies showed elevated white blood cell count (19.5 thousand/µL), with 75% neutrophils and no bands.

**Figure 1:**
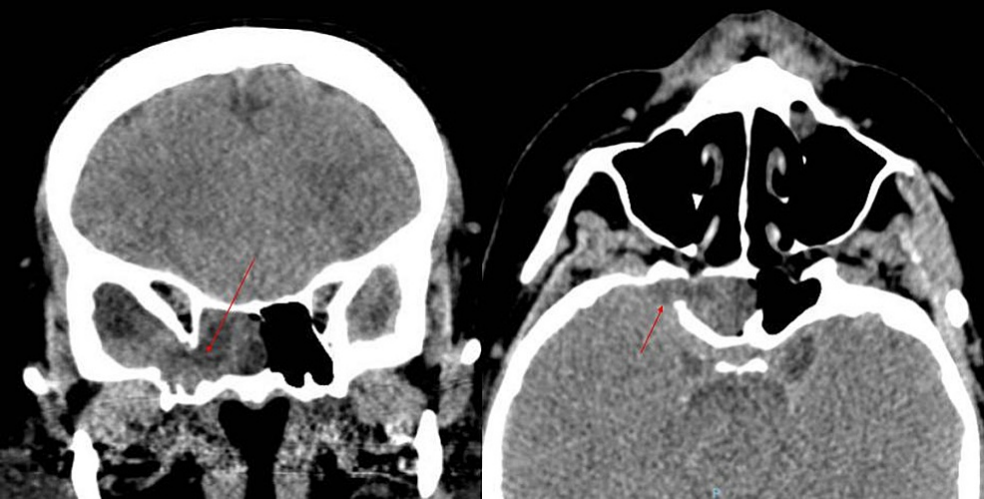
Head computed tomographic scan shows a 1.1 cm osseous defect at the lateral wall of a right sphenoid sinus (red arrow), resulting in direct communication between the middle cranial fossa and the right sphenoid sinus Coronal (left), axial (right)

**Figure 2:**
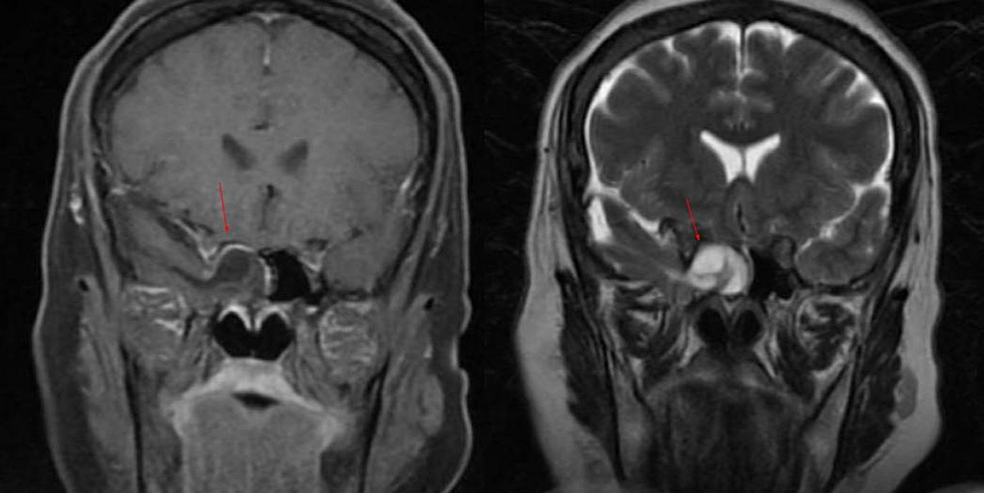
Brain magnetic resonance imaging, coronal T1 with gadolinium contrast (left), coronal T2 (right) demonstrating herniation of the meninges and portions of the inferomedial right temporal lobe through the defect into the right sphenoid sinus (red arrow)

Head computed tomographic (CT) scan showed a 1.1 cm osseous defect at the lateral wall of a right sphenoid sinus, resulting in direct communication between the middle cranial fossa and the right sphenoid sinus (Figure 1). Brain magnetic resonance imaging (MRI) demonstrated herniation of the meninges and portions of the inferomedial right temporal lobe through a defect in the lateral wall of the right sphenoid sinus, extending to the sphenoethmoidal recess and posterior right ethmoid air cells (Figure 2). The study showed no evidence of an empty sella or obliteration of venous dural sinuses. A lumbar puncture was performed for suspected meningitis, which revealed a low glucose level (34 mg/dL) and an elevated white blood cell count (281 cells/µL). The opening pressure was 12 cm H_2_O.

The patient was started on intravenous antibiotics consisting of vancomycin, ampicillin, and ceftriaxone. The pneumococcal vaccine was also given. The patient was operated on intracranially using a right pterional craniotomy. The dura mater was opened, and the temporal lobe was gently elevated to expose the portion of the temporal lobe herniated through the bone defect at the lateral wall of the sphenoid sinus. A dural dissection was performed around the bone defect, and the herniated part of the right temporal lobe, and its associated meninges, were excised entirely. The right sphenoid sinus defect was packed with autologous abdominal fat. The bone defect was intracranially sealed off with a double-layer technique, consisting of two layers of collagen dural substitute topped with fibrin sealant.

**Figure 3:**
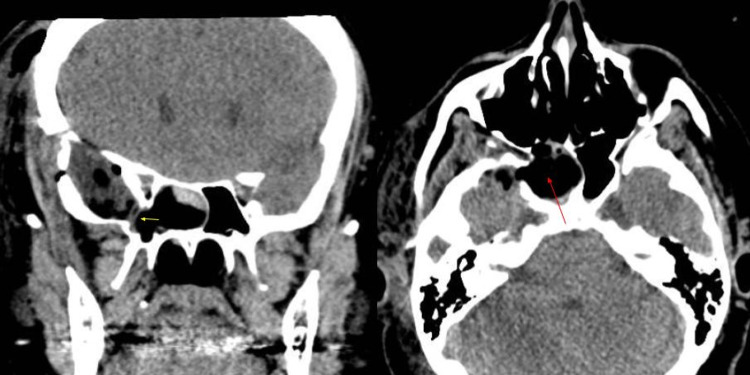
Head computed tomographic scan after surgical correction via a right pterional craniotomy with postsurgical changes in the anterior temporal lobe and a small amount of pneumocephalus. Fat graft has been placed inside the right sphenoid sinus (red arrow). The defect had been covered with a collagen dural substitute (yellow arrow) Coronal (left), axial (right)

After surgical correction, the postoperative brain CT scan showed postsurgical changes at the anterior temporal lobe and a small pneumocephalus (Figure 3). The autologous fat graft was identified inside the right sphenoid sinus. The bone defect had been covered with a collagen dural substitute. The resected specimen consisted of a gelatinous tissue measuring 2.5 cm x 2 cm x 0.5 cm.

**Figure 4:**
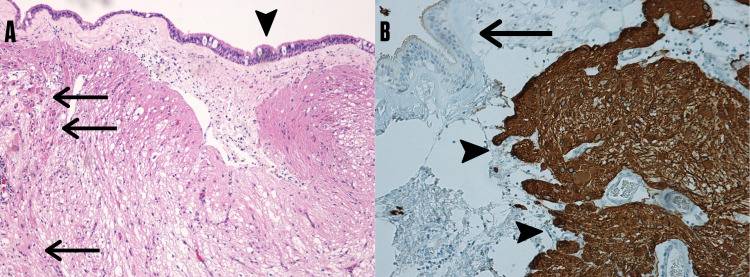
Histopathological examination of the resected meningoencephalocele (A) Neuroglial tissue with "gemistocytic type" astrocytes (arrows) lined by ciliated pseudostratified columnar epithelium (arrowhead) (Hematoxylin and Eosin staining, x100); (B) Glial fibrillary acidic protein immunohistochemistry staining highlights the glial tissue (arrowheads) while there is no reaction in the surrounding soft tissue and respiratory epithelium (arrow) (x200).

The microscopic histopathological examination showed normal brain parenchyma with mature astrocytes and few gemistocytic astrocytes partially lined by ciliated pseudostratified columnar epithelium (respiratory epithelium). Glial fibrillary acidic protein immunohistochemistry staining confirmed the presence of glial tissue in the specimen, with no reaction in the surrounding soft tissue and respiratory epithelium (Figure 4).

The patient remained asymptomatic with down-trending leukocytosis while completing the antibiotic regimen. The cerebrospinal fluid (CSF) culture was negative. The patient has not had any recurrence of rhinorrhea after a six-month follow-up period. Headaches resolved completely. After surgery, she has hypoesthesia at the right maxillary divisions of the trigeminal nerve. This finding was caused by the proximity of the trigeminal nerve to the dural dissection that we performed at the bone defect, which was just lateral to the lateral wall of the cavernous sinus wall and foramen rotundum. She did not complain of a dry eye, proving the integrity of the vidian nerve inside the sphenoid sinus.

## Discussion

Meningoencephaloceles are herniation of the meninges and parts of the brain through defects in the cranial vault. Spontaneous anterior and middle cranial fossa meningoencephalocele had a prevalence of 0.0074%, with a slight female predominance [1]. This report presented a morbidly obese female with a delayed diagnosis of a lateral wall sphenoid sinus meningoencephalocele. She was misdiagnosed on multiple occasions with chronic sinusitis and nasal polyps.

Obesity has been a risk factor to both the dehiscence of the skull base and the creation of CSF fistulae, as it leads to increased intra-abdominal and intracranial pressures by decreasing cerebral venous return to the heart [2]. Failure of the normal ossification of the sphenoid sinus can produce abnormalities in the bony walls of the sinus. Changes in CSF pressures, prominent arachnoid granulations due to impaired CSF reabsorption, and pneumatization of the sinuses may all potentiate osseous dehiscence throughout one’s lifetime. Some patients may not initially present idiopathic intracranial hypertension, as they are decompressed by the leak, only becoming symptomatic after repairing the defect [2]. Idiopathic intracranial hypertension, evidenced by arachnoid pits causing erosion of the sphenoid wall, is thought to be the etiology of a lateral sphenoid meningoencephalocele [3].

Depending on the location of these herniations, meningoencephaloceles may lead to rhinorrhea, otorrhea, middle ear effusions, recurrent ear infections, progressive hearing loss, facial nerve paresis, pneumocephalus, encephalitis, meningitis, intracranial abscesses, temporal lobe epilepsy, and aphasia. About 10% of the patients have epileptic seizures [4]. However, in symptomatic cases, a CSF leak is the most common clinical manifestation [5]. Some cases can present with trigeminal nerve involvement, as the bony defect in the greater sphenoid wing is lateral to the foramen rotundum [6]. Soyer et al. reported a sphenoid meningoencephalocele producing paresthesia in the trigeminal nerve’s territory with diminished sensation in the right mandibular nerve [7].

Radiographic evaluation with head CT scan, brain MRI, and MRI cisternography is usually effective in delineating the skull base defect [8]. If defects are not found but high suspicion still exists, surgical exploration with preoperative intrathecal fluorescein may be sought. The sphenoid sinus’s lateral wall is the most common site of meningoencephalocele and spontaneous CSF leak. The defect’s location is in the lateral recess of the sphenoid sinus, laterally to the mandibular nerve course. The herniated tissue can extend to the ethmoidal sinuses or the pterygopalatine fossa. Meningoencephalocele through the foramen rotundum had been reported but without trigeminal nerve symptoms [9-10].

Due to the high risk for morbidity and mortality, urgent management of symptomatic meningoencephaloceles is highly recommended. The use of preoperative and postoperative lumbar drain placement is controversial and has an unclear overall benefit. There is currently no consensus for an optimal surgical approach to the management of meningoencephaloceles. Pterional, temporal, subtemporal craniotomies, and endoscopic endonasal approaches may all be appropriate options for managing middle fossa meningoencephaloceles. Endoscopic surgery has comparable success rates but lower morbidity than intracranial procedures. Endoscopic surgery uses an expanded endonasal approach with the drilling of the pterygoid process and a nasoseptal flap [11]. Transpetrygoid is the most utilized approach, accounting for almost half of the cases reported in the literature [8]. The maxillary nerve is partially or totally visualized in this approach. Repair is made using a multilayered repair with allograft and abdominal fat covered with a nasoseptal flap. The success rate for endoscopic repair of lateral recess leaks varies between 60% and 100% [6,12-14]. Recurrence of these defects has been estimated to occur in 20% of the cases [15]. Therefore, follow-up is critical to detect recurrent leakage.

Predictable sequelae from the endoscopic approach may be trigeminal paresthesia from manipulating the maxillary nerve and occasional eye dryness from vidian nerve manipulation. Neural injuries of the mandibular branch of the trigeminal nerve, vidian nerve, and greater palatine nerve are usually transient [8]. Most patients reported that these symptoms were not bothersome or life-altering [14]. Only eight literature reports have described hypoesthesia or paresthesia of the mandibular nerve after endoscopic resection of a lateral sphenoid meningoencephalocele [5,12-14,16-19]. Numbness was more common than paresthesias in those cases [14]. No previous case of paresthesia or numbness has been described after intracranial surgery. Our patient developed hypoesthesia at the right maxillary division of the trigeminal nerve after the repair. This finding was caused by the proximity of the trigeminal nerve to the dural dissection that we performed at the bone defect, which was just lateral to the lateral wall of the cavernous sinus wall and foramen rotundum. Patients should be counseled that injury to the trigeminal nerve can occur during the repair of a meningoencephalocele at the lateral wall of the sphenoid sinus.

## Conclusions

Repair of meningoencephalocele can be performed by an endoscopically endonasal approach or by a transcranial approach. The intracranial repair should involve a layered technique in which the cranial defect is covered with allograft material, with fat obliterating the sphenoid wall defect. Transient or permanent injury to the trigeminal nerve can occur with either procedure. Close follow-up is necessary, as recurrences may occur secondary to idiopathic intracranial hypertension.
